# Assessment of the Concordance and Diagnostic Accuracy Between Elecsys and Lumipulse Fully Automated Platforms and Innotest

**DOI:** 10.3389/fnagi.2021.604119

**Published:** 2021-03-04

**Authors:** Farida Dakterzada, Ricard López-Ortega, Alfonso Arias, Iolanda Riba-Llena, Maria Ruiz-Julián, Raquel Huerto, Nuria Tahan, Gerard Piñol-Ripoll

**Affiliations:** Cognitive Disorders Unit, Clinical Neuroscience Research Group, Santa Maria University Hospital, IRBLleida, Lleida, Spain

**Keywords:** Alzheimer’s disease, assay automation, biomarker, cerebrospinal fluid, cut-off

## Abstract

Manual ELISA assays are the most commonly used methods for quantification of biomarkers; however, they often show inter- and intra-laboratory variability that limits their wide use. Here, we compared the Innotest ELISA method with two fully automated platforms (Lumipulse and Elecsys) to determine whether these new methods can provide effective substitutes for ELISA assays. We included 149 patients with AD (*n* = 34), MCI (*n* = 94) and non-AD dementias (*n* = 21). Aβ42, T-tau, and P-tau were quantified using the ELISA method (Innotest, Fujirebio Europe), CLEIA method on a Lumipulse G600II (Fujirebio Diagnostics), and ECLIA method on a Cobas e 601 (Roche Diagnostics) instrument. We found a high correlation between the three methods, although there were systematic differences between biomarker values measured by each method. Both Lumipulse and Elecsys methods were highly concordant with clinical diagnoses, and the combination of Lumipulse Aβ42 and P-tau had the highest discriminating power (AUC 0.915, 95% CI 0.822–1.000). We also assessed the agreement of AT(N) classification for each method with AD diagnosis. Although differences were not significant, the use of Aβ42/Aβ40 ratio instead of Aβ42 alone in AT(N) classification enhanced the diagnostic accuracy (AUC 0.798, 95% CI 0.649–0.947 vs. AUC 0.778, 95% CI 0.617–0.939). We determined the cut-offs for the Lumipulse and Elecsys assays based on the Aβ42/Aβ40 ratio ± status as a marker of amyloid pathology, and these cut-offs were consistent with those recommended by manufacturers, which had been determined based on visual amyloid PET imaging or diagnostic accuracy. Finally, the biomarker ratios (P-tau/Aβ42 and T-tau/Aβ42) were more consistent with the Aβ42/Aβ40 ratio for both Lumipulse and Elecsys methods, and Elecsys P-tau/Aβ42 had the highest consistency with amyloid pathology (AUC 0.994, 95% CI 0.986–1.000 and OPA 96.4%) at the ≥0.024 cut-off. The Lumipulse and Elecsys cerebrospinal fluid (CSF) AD assays showed high analytical and clinical performances. As both automated platforms were standardized for reference samples, their use is recommended for the measurement of CSF AD biomarkers compared with unstandardized manual methods, such as Innotest ELISA, that have demonstrated a high inter and intra-laboratory variability.

## Introduction

Alzheimer’s disease (AD) is the most prevalent age-related neurodegenerative disease, accounting for 60–80% of cases of dementia. The extracellular amyloid plaques arising from the accumulation of amyloid β42 protein (Aβ42) and intracellular neurofibrillary tangles formed by aggregations of hyperphosphorylated tau protein (P-tau) are the two main pathological hallmarks of AD (Serrano-Pozo et al., 2011). Both of these pathological characteristics are specific to AD, while neurodegeneration, characterized by an increase in total-tau protein (T-tau), is a non-specific biomarker that can be caused by several neurodegenerative diseases (Jack et al., 2018). Aβ42, P-tau, and T-tau are considered core AD biomarkers that can be measured in cerebrospinal fluid (CSF). Their use increases the accuracy of the diagnosis and prediction of the progression from mild cognitive impairment (MCI) to AD and can differentiate between AD and other causes of dementia or neuropsychiatric problems (Albert et al., 2011; McKhann et al., 2011; Sperling et al., 2011). In addition, the inclusion of these biomarkers in diagnosis benefits populations included in clinical trials (Jack et al., 2018).

Currently, enzyme-linked immunoassay (ELISA) is the most widely used approach for the detection of AD core biomarkers in CSF. However, these ELISA methods often show considerable inter and intra-lab variability that prevents the use of standard cut-off values and precludes the wide use of CSF biomarkers in clinical practice. To circumvent this problem, Fujirebio Diagnostics and Roche Diagnostics have recently developed fully automated platforms for the analysis of CSF biomarkers. Fujirebio has implemented four CSF analytes (Aβ42, Aβ40, T-tau, and P-tau) on the fully automated Lumipulse G System. The measurement method is based on a two-step sandwich chemiluminescent enzyme-immunoassay (CLEIA). The Lumipulse Aβ42 assay is standardized according to certified reference material (CRM) developed by the International Federation of Clinical Chemistry and Laboratory Medicine (IFCC) and the Joint Research Centre (JRC). These platforms consist of three CRMs based on human CSF, with low, middle and high concentrations of Aβ42. However, fully automated Elecsys assays for CSF Aβ42, T-tau and P-tau are run on Elecsys and *Cobas e* immunoassay analyzers (Roche Diagnostics GmbH, Penzberg, Germany). The measurement is performed based on the electrochemiluminescence immunoassay (ECLIA) in a two-step sandwich assay. The Elecsys Aβ42 assay has been standardized by a Joint Committee for Traceability in Laboratory Medicine (JCTLM) with an approved reference measurement procedure (RMP). Therefore, all assay lots are standardized to a sample set with target values derived from liquid chromatography–tandem mass spectrometry (LC-MS/MS) (Leinenbach et al., 2014).

Previous studies have evaluated the consistency between each of these automated methods with manual ELISA methods or Amyloid PET imaging (Janelidze et al., 2017; Hansson et al., 2018; Kollhoff et al., 2018; Schindler et al., 2018; Willemse et al., 2018; Alcolea et al., 2019; Bayart et al., 2019; Zecca et al., 2019; Kaplow et al., 2020). However, there are no studies that have compared the efficacy of Innotest, Lumipulse and Elecsys methods in a single cohort of patients.

The aims of this study were (a) to assess the concordance between core AD biomarkers measured in CSF using Innotest, Lumipulse and Elecsys methods; (b) to evaluate the diagnostic accuracy of biomarkers and their ratios measured by each method; (c) to assess the discriminating power of AT(N) groups that were generated by the results of the different biomarkers for each of these three technologies and (d) to define the CSF cut-off points for both Lumipulse and Elecsys assays based on the Lumipulse Aβ42/40 status.

## Materials and Methods

### Study Population

A total of 149 patients [AD (*n* = 34), MCI (*n* = 94) and non-AD dementias (*n* = 21)] were included in this study. The study population was recruited consecutively between July 2018 and July 2019 from patients attending the Cognitive Disorders Unit at the Hospital Universitari Santa Maria (Lleida, Spain). Inclusion criteria comprised presentation with suspected cognitive dysfunction at the memory clinic, for which the neurologist requested CSF analysis. The diagnosis of probable AD and MCI was performed based on NIAA criteria (Albert et al., 2011; McKhann et al., 2011). Each non-AD patient fulfilled the criteria for the specific diagnostic criteria of the disorder considered (e.g., Fronto-temporal dementia, Lewy body dementia, etc.) (Gorno-Tempini et al., 2011; Rascovsky et al., 2011; McKeith et al., 2017). The included patients signed an internal regulatory document stating that residual samples used for diagnostic procedures could be used for research studies without any additional informed consent.

### CSF Collection and Storage

Cerebrospinal fluid samples were collected between 8 a.m. and 10 a.m. after an overnight fast into 10-mL polypropylene tubes (Sarstedt, 62.610.201). The tubes were inverted several times, and the CSF was processed based on the recommendations provided by each manufacturer. For the Lumipulse assay, the samples were centrifuged at 2,000 × *g* for 10 min at room temperature and aliquoted into two 2-mL polypropylene tubes (Sarstedt, 72.694.007), with each tube containing 1 mL of CSF. For the Elecsys method, the samples were aliquoted into two 0.5-mL polypropylene tubes (Sarstedt 72.730.005) after centrifugation. For the Innotest assay, the CSF was aliquoted into two 2-mL polypropylene tubes (Sarstedt, 72.694.007) after centrifugation. The samples were stored at −80°C until analyses.

### CSF Analysis

Measurements of Aβ42 and Aβ40 (only for lumipulse), T-tau, and P-tau were performed at the clinical laboratory of Hospital Universitari Arnau de Vilanova, Lleida. On the day of the analysis, samples were thawed at room temperature, and the tubes were vortexed briefly. The biomarkers were measured directly from the storage tube and in five separate batches for all three methods. For each method, the same batch of reagents was used for each biomarker throughout the study, and for each sample, all analytes were quantified in the same run and from the same aliquot. For the ELISA method, Innotest Aβ42, Innotest htau-Ag, and Innotest P-tau (181P) assays (Fujirebio, Europe) were used. Innotest calibrator concentrations ranged from 63 to 4000 pg/mL for Aβ42, 40 to 2300 pg/mL for T-tau, and 16 to 1000 pg/mL for P-tau. According to previous analyses in clinical practice, cut-offs at our center were determined to be <600 pg/mL for Aβ42, >425 pg/mL for T-tau, and >65 pg/mL for P-tau. For the ECLIA method, the tubes analyzed using the Elecsys Aβ42 CSF, Elecsys T-tau CSF, and Elecsys P-tau (181P) CSF assays (Roche Diagnostics GmbH) were run on the cobas e 601 analyzer (Roche Diagnostics) per the manufacturer’s instructions. Elecsys measuring ranges were as follows: 200 to 1700 pg/mL for Aβ42, 80 to 1300 pg/mL for T-tau, and 8 to 120 pg/mL for P-tau. For data analysis, we used the cut-offs recommended by the manufacturer, which were as follows: ≤1000 pg/mL for Aβ42, >300 pg/mL for T-tau, and >27 pg/mL for P-tau. Seventeen samples had Aβ42 levels above the upper limit of the measuring range (1700 pg/mL) and were eliminated from the analysis. The results of the Elecsys Aβ42 assay were standardized to the JCTLM-approved RMP for quantitation of Aβ42 in human CSF, based on LC-MS/MS (Leinenbach et al., 2014). For the CLEIA technology, the CSF biomarkers were quantified using the Lumipulse Aβ42, Aβ40, T-tau, and P-tau (181P) assays on the LUMIPULSE G600II automated platform (Fujirebio) per the manufacturer’s instructions. Lumipulse measuring ranges were 9–2,335 pg/mL for Aβ42, 150–2,000 pg/mL for T-tau, and 1.1–400 pg/mL for p-Tau. The following cut-offs that had been determined by Fujirebio were used for data analysis: Aβ42 < 600 pg/mL, Aβ42/40 < 0.069, T-tau > 400 pg/mL, and P-tau > 56.5 pg/mL. The results of the Lumipulse Aβ42 presented in this work have been standardized with CRMs developed by the IFCC and JRC (Kuhlmann et al., 2017). The personnel involved in the CSF analyses were blind to the clinical diagnosis.

### Statistical Analyses

All statistical analyses were performed using IBM SPSS version 25 (Armonk, NY, United States). One-way ANOVA and Chi-square tests were used for analysis of quantitative and qualitative variables, respectively. The quantitative variables were presented as means (±standard deviation, SD), and the qualitative variables were presented as percentages. To evaluate the correlation between methods, we used Pearson’s correlation coefficient (r), paired *t*-tests for paired samples, and the Bland-Altman plot. The diagnostic accuracy of the biomarkers/AT(N) classification was analyzed using a binary logistic regression model. In this model, the sensitivity was defined as the percentage of correct classification of AD diagnosis and the specificity as the percentage of correct classification of non-AD dementias diagnosis. Furthermore, we used this statistical model to evaluate the predictive value of the biomarkers with respect to AD prognosis. The receiver operating characteristic (ROC) analysis for evaluating diagnostic accuracy was further analyzed using the Hanley and McNeil method (Hanley and McNeil, 1982) to compare the Area Under the Curve (AUC). Values of |z| ≥ 1.96 were considered evidence that the true ROC areas were different. We also performed ROC analysis to determine the cut-offs for the core AD biomarkers and the ratios that best distinguished Lumipulse Aβ42/40+ individuals. In addition, the cut-offs were also determined based on the Innotest Aβ42 status. We determined the positive percent agreement (PPA) and negative percent agreement (NPA), and the single analyte value (or ratio) with the highest Youden index (PPA + NPA – 1) was identified as the cut-off value. Overall percent agreement (OPA) was defined as the sum of the Aβ42/40 + individuals who were positive for a CSF biomarker measure and the Aβ42/40 − individuals who were negative for a CSF biomarker measure divided by the cohort size, thereby providing an estimate of accuracy.

## Results

### Patient Characteristics

The demographic characteristics and biomarker results are summarized in Table 1. The average age of participants was 74 years, and 55% were female. Syndrome diagnoses in the cohort were the following: 34 (22.8%) with AD, 94 (63.1%) with MCI, and 21 (14.1%) with non-AD dementia. There were no significant differences between diagnostic groups for demographic and clinical variables except for MMSE score and hypertension (*P* < 0.0001 and *P* < 0.05, respectively). The mean MMSE score was lower [19.6 (4.2 SD)] for AD patients compared with the two other groups, followed by non-AD dementia patients [21.9 (4.6 SD)] and MCI subjects [25.2 (3.1 SD)]. For all three assays, all CSF biomarker concentrations were significantly different between the three diagnostic groups, except Lumipulse Aβ40 (*P* > 0.05). For Elecsys, samples that had Aβ42 values above the upper limit of detection (1700 pg/mL) were omitted from analysis (*n* = 17 samples, MCI 11, AD 4, and 2 non-AD patients).

**TABLE 1:** The demographic characteristics and biomarker results for AD, MCI, and non-AD patients.

	**All participants**	**AD**	**MCI**	**Non-AD dementia**	***P-*value**
*n* (%)	149 (100%)	34 (22.8%)	94 (63.1%)	21 (14.1%)	
Age (years)	73.82 (6.85)	74.00 (8.78)	73.86 (6.05)	73.33 (7.07)	0.937
Sex (% female)	55.7%	67.6%	54.3%	42.9%	0.178
MMSE score	23.41 (4.29)	19.62 (4.24)	25.16 (3.07)	21.90 (4.57)	<0.0001
Family history of cognitive impairment	28.9%	23.5%	29.8%	33.3%	0.7
Hypertension	57.7%	67.6%	55.3%	52.4%	0.003
Diabetes Mellitus	20.1%	26.5%	19.1%	14.3%	0.509
Dyslipidemia	44.3%	47.1%	44.7%	38.1%	0.803
Depression	35.6%	29.4%	36.2%	42.9%	0.587
**Innotest**					
Aβ42 pg/mL	581.37 (247.46)	405.65 (115.36)	614.11 (252.18)	722.48 (239.53)	<0.0001
T-tau pg/mL	507.85 (354.32)	684.47 (393.01)	450.71 (277.00)	477.67 (498.05)	0.003
P-tau pg/mL	67.55 (28.62)	83.81 (34.75)	64.68 (25.10)	53.91 (21.14)	<0.0001
T-tau/Aβ42	1.130 (1.092)	1.908 (1.623)	0.935 (0.743)	0.727 (0.687)	<0.0001
P-tau/Aβ42	0.148 (0.116)	0.234 (0.167)	0.130 (0.083)	0.088 (0.056)	<0.0001
**Elecsys**					
Aβ42 pg/mL	770.69 (363.12)	572.50 (179.97)	807.75 (369.95)	970.04 (433.81)	<0.0001
T-tau pg/mL	287.80 (155.38)	379.65 (188.44)	261.39 (118.65)	251.37 (189.67)	<0.0001
P-tau pg/mL	27.38 (17.17)	38.92 (23.19)	24.73 (13.22)	19.56 (11.71)	<0.0001
T-tau/Aβ42	0.463 (0.338)	0.719 (0.470)	0.390 (0.225)	0.318 (0.242)	<0.0001
P-tau/Aβ42	0.045 (0.040)	0.075 (0.060)	0.038 (0.024)	0.027 (0.022)	<0.0001
**Lumipulse**					
Aβ42 pg/mL	571.43 (276.75)	415.28 (119.15)	599.55 (289.63)	698.41 (301.96)	<0.0001
Aβ40 pg/mL	10317.68 (3339.78)	10597.44 (3605.39)	10363.14 (3111.28)	9661.24 (3935.09)	0.59
Aβ42/40	0.056 (0.022)	0.041 (0.010)	0.058 (0.022)	0.073 (0.022)	<0.0001
T-tau pg/mL	510.37 (356.34)	731.85 (404.16)	438.84 (256.48)	471.95 (505.21)	<0.0001
P-tau pg/mL	81.50 (56.72)	122.96 (73.80)	72.76 (44.18)	53.49 (40.49)	<0.0001
T-tau/Aβ42	1.144 (1.037)	1.933 (1.504)	0.940 (0.700)	0.779 (0.740)	<0.0001
P-tau/Aβ42	0.190 (0.190)	0.330 (0.291)	0.159 (0.123)	0.101 (0.101)	<0.0001

*Unless otherwise specified, results are presented as mean (standard deviation). MMSE, Mini-mental state examination; AD, Alzheimer’s disease; MCI, mild cognitive impairment; non-AD dementia, non-Alzheimer’s disease dementia. *P*-values were calculated by comparing AD, MCI, and non-AD dementia participants using one way ANOVA for continuous variables and Pearson Chi2 for categorical variables.*

### Concordance Between Innotest and Lumipulse Assays

**FIGURE 1:**
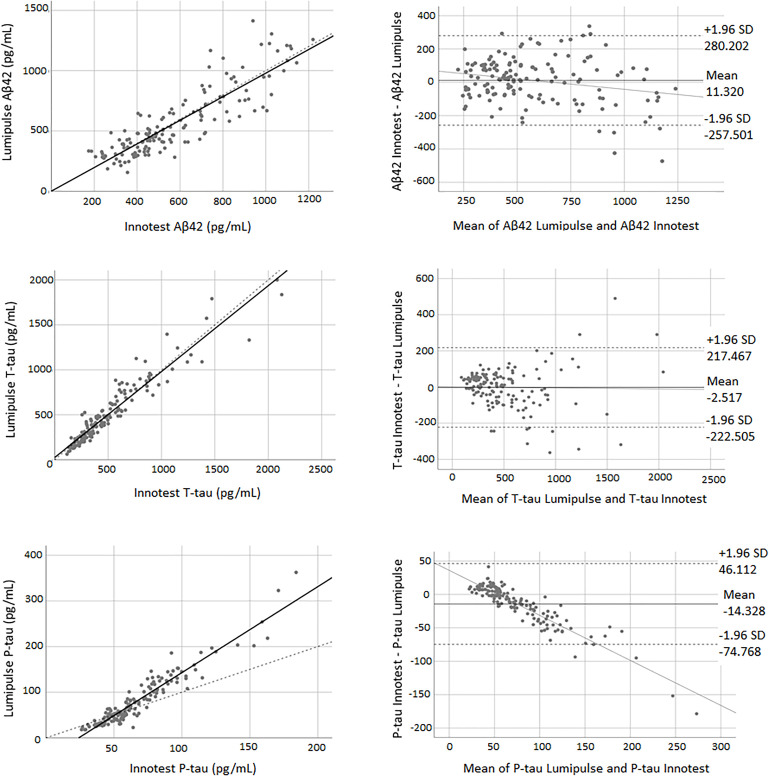
The correlation and Bland-Altman plots for Aβ42, T-tau, and P-tau measurements obtained by Lumipulse and Innotest ELISA methods (*n* = 149). Each point is defined as the measurements of Lumipulse and ELISA assays on the same biological sample. In correlation plots, the solid lines represent the estimated regression line, and the dotted line represents the identity line (*x* = *y*). In the Bland-Altman plots, solid lines represent the slope observed.

Pearson’s correlations indicated a high correlation between biomarkers of both methods. Figure 1 shows the correlation and Bland-Altman plots for biomarkers quantified by Innotest and Lumipulse. The correlation coefficient between the two methods was 0.87 for Aβ42 (*P* < 0.0001), 0.95 for T-tau (*P* < 0.0001) and 0.95 for P-tau (*P* < 0.0001). The concordance between the values of the biomarkers between the two methods was assessed using paired sample *t*-tests. Our results indicated that there was high consistency in the Aβ42 (observed slope 0.98, *t*-test *p* = 0.319) and T-tau (observed slope 0.96, *t*-test *P* = 0.785) values between the two methods (Figure 1). Lumipulse Aβ42 values were slightly higher than those for Innotest, while Lumipulse T-tau values were slightly lower than those for Innotest; however, these differences were not statistically significant (Figure 1). As shown in the Bland-Altman plot, the bias (mean of the differences) for Aβ42 was 11.320 units (pg/mL) (continuous line) between the two methods. The regression line for the differences indicated that there was a non-significant negative trend in the differences as the magnitude of the measured variable increased. For T-tau, there was a bias of −2.157 units between the two methods. However, the results of Lumipulse and Innotest were not consistent with respect to P-tau values (observed slope 1.884, *t*-test *P* < 0.0001). For P-tau, there was a bias of −14.328 units (continuous line). The regression line for the differences indicated that there was a systematic proportional bias between the values of the two methods with a negative trend in the differences as the magnitude of P-tau values increased, especially for values greater than 50 pg/mL. Among all assays evaluated, approximately 95% of the measured values were within ±1.96 SD of the bias (Figure 1).

### Concordance Between Innotest and Elecsys Assays

**FIGURE 2:**
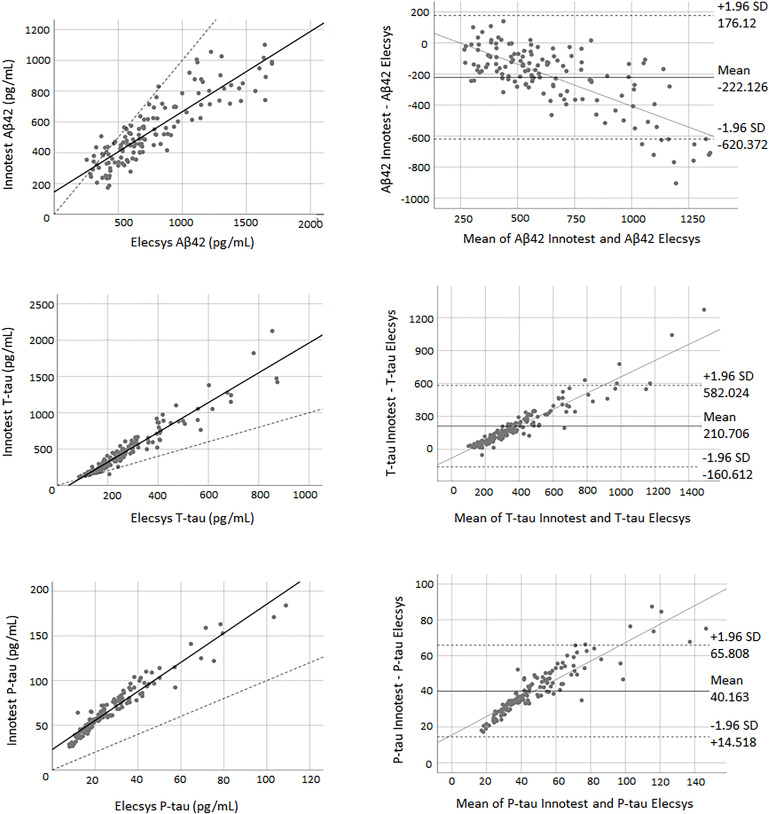
The correlation and Bland-Altman plots for Aβ42, T-tau, and P-tau measurements obtained by Elecsys and Innotest methods (for P-tau and T-tau *n* = 145; for Aβ42 *n* = 135). Each point is defined as the measurement of Elecsys and Innotest assays on the same biological sample. In correlation plots, the solid lines represent the estimated regression line, and the dotted line represents the identity line (*x* = *y*). In the Bland-Altman plots, solid lines represent the slope observed.

Pearson’s correlations indicated a high correlation between biomarkers of both methods. The correlation coefficient between the two methods was 0.88 for Aβ42 (*P* < 0.0001), 0.96 for T-tau (*P* < 0.0001) and 0.97 for P-tau (*P* < 0.0001). The paired samples *t*-test demonstrated that there was weak concordance between the two methods for all of the biomarkers. For all three biomarkers, the adjustment line (continuous line) (observed slope of 0.52 for Aβ42, *t*-test *P* < 0.0001; observed slope of 2.05 for T-tau, *t*-test *P* < 0.0001; and observed slope of 1.62 for P-tau, *t*-test *P* < 0.0001) was significantly separated from the perfect agreement line (dashed line) (Figure 2). The Bland-Altman plot indicated that there was a bias of −222.13 units (continuous line) between the two methods for Aβ42 (i.e., the Elecsys method quantified on average 222.13 pg/mL more Aβ42 than the Innotest assay). The regression line demonstrated a proportional systematic bias with a negative trend of differences as the magnitude of Aβ42 increased. For T-tau and P-tau, the biases (mean of differences) were 210.70 and 40.16 units, respectively. The regression line of the differences indicated a proportional systematic bias for both biomarkers with a positive trend of differences as the magnitude of these biomarkers increased. For all assays evaluated, approximately 95% of measured values were within ±1.96 SD of the bias (Figure 2).

### Concordance Between Elecsys and Lumipulse Assays

**FIGURE 3:**
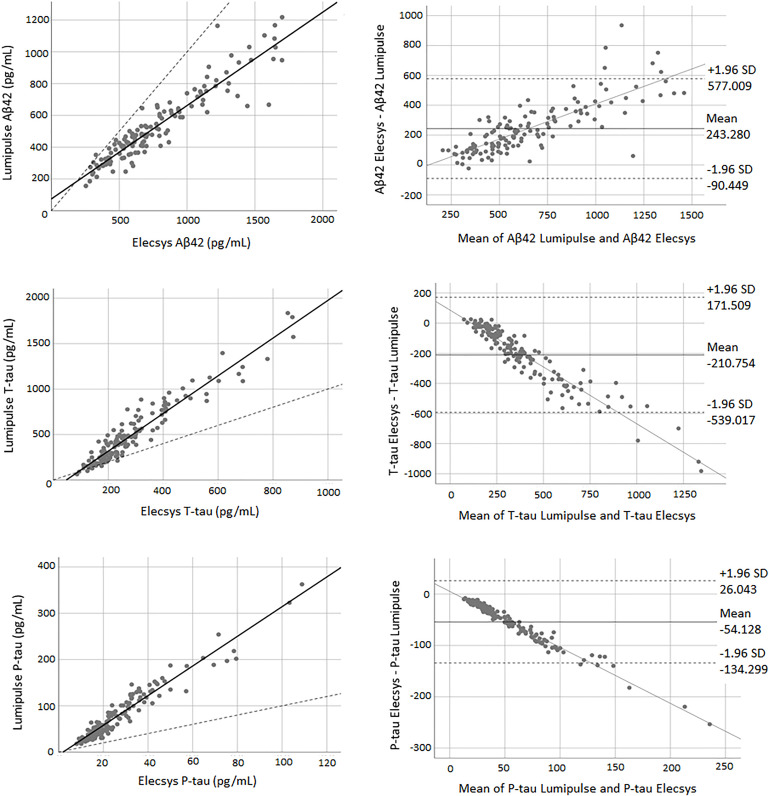
The correlation and Bland-Altman plots for Aβ42, T-tau, and P-tau measurements obtained by Lumipulse and Elecsys methods (for P-tau and T-tau *n* = 145; for Aβ42 *n* = 137). Each point is defined as the measurement of Lumipulse and Elecsys assays on the same biological sample. In correlation plots, the solid lines represent the estimated regression line, and the dotted line represents the identity line (*x* = *y*). In the Bland-Altman plots, solid lines represent the slope observed.

There was a high correlation between all three biomarkers for both methods. The correlation coefficient between the two methods was 0.94 for Aβ42 (*P* < 0.0001), 0.95 for T-tau (*P* < 0.0001), and 0.96 for P-tau (*P* < 0.0001). Figure 3 shows the correlation and Bland-Altman plots for biomarkers quantified by Elecsys and Lumipulse. The *t*-test results indicated that there was weak concordance between all pairs of biomarkers (*P* < 0.0001). For all three biomarkers, the adjustment line (continuous line) (observed slope of 0.59 for Aβ42, *t*-test *P* < 0.0001; observed slope of 2.07 for T-tau, *t*-test *P* < 0.0001; and observed slope 3.21 for P-tau, *t*-test *P* < 0.0001) was significantly separated from the perfect agreement line (dashed line) (Figure 3). The Bland-Altman plot indicated that there was a bias of 243.28 (continuous line) for Aβ42, meaning that Lumipulse quantified 243.28 pg/mL less Aβ42 on average than Elecsys. The regression line demonstrated a proportional systemic bias with a positive trend of differences as the magnitude of Aβ42 increased. For T-tau and P-tau, the biases between the two assays were −210.754 and −54.128 units, respectively. The regression line of the differences indicated a proportional systematic bias for both biomarkers with a negative trend of differences as the magnitude of these biomarkers increased. For all assays evaluated, approximately 95% of measured values were within ± 1.96 SD of the bias (Figure 3).

### AD Diagnostic Accuracy of the Biomarkers Quantified by Each Method

Using binary logistic regression, we evaluated the diagnostic accuracy of biomarkers quantified by each assay (clinical diagnosis is generally considered the gold standard). To discriminate AD from non-AD patients, the combined use of Aβ42 and P-tau was the best approach for all three assays. The Aβ42/40 ratio of Lumipulse also had high discriminating power, comparable with the combined use of Aβ42 and P-tau, to differentiate between the two diagnostic groups (AUC 0.882, 95% CI 0.785–0.980). Among all three methods, Lumipulse Aβ42 and P-tau had higher discriminating power with an AUC of 0.915 (95% CI 0.822–1.000). This combination of biomarkers had 91.2% sensitivity and 76.2% specificity for a correct classification of diagnostic groups, and their predictive accuracy was estimated to be 85.5%. However, the AUCs were not significantly different between the three methods, as they were assessed using the Hanley and McNeil method (|z| < 1.96). However, the sensitivity, specificity and predictive accuracy slightly differed between methods (Table 2).

**TABLE 2:** Biomarkers with the best discriminating power between AD and non-AD dementia patients.

	**Biomarker**	**AUC (95% CI)**	**Sensitivity**	**Specificity**	Total% of predictive accuracy*	*z-*value**
Lumipulse	Aβ42 + P-tau	0.915 (0.822–1.000)	91.2%	76.2%	85.5%	*z* = 0.997 vs. Lumipulse Aβ42/40; *z* = 0.639 vs. Innotest; *z* = 1.673 vs. Elecsys
	Aβ42/40	0.882 (0.785–0.980)	94.1%	71.4%	85.5%	*z* = −0.394 vs. Innotest; *z* = 0.033 vs. Elecsys
Innotest	Aβ42 + P-tau	0.895 (0.801–0.989)	94.1%	76.2%	87.3%	*z* = 0.544 vs. Elecsys
Elecsys	Aβ42 + P-tau	0.881 (0.774–0.988)	91.1%	72.2%	84.6%	

AUC, Area under the curve.

*The percentage of correct classification of AD + correct classification of non-AD/all cases.

**Values of |z| < 1.96 were taken as evidence that the true ROC areas were not different.

### Diagnostic Accuracy of the AT(N) Classification for Each Method

The same statistical model was used to evaluate the discriminating power of the AT(N) classification for each method. We classified our study population into 6 AT(N) (0, 1, 2, 3, 4, and 5) groups based on the results of the three core AD biomarkers (Jack et al., 2018). Biomarkers were grouped into those for β amyloid deposition, pathologic tau, and neurodegeneration [AT(N)]. Here, A referred to levels of Aβ42 (Aβ42/40) in CSF, T referred to levels of P-tau in CSF, and (N) referred to levels of T-tau in CSF. We provided two AT(N) classifications for Lumipulse, one based on the results of Aβ42, T-tau and P-tau and the other one based on the Aβ42/40, T-tau and P-tau values (Table 3). Patients who were grouped as AT(N) 0 were negative for all three biomarkers. Patients in the AT(N) 1 group were only positive for Aβ42 or the Aβ42/40 ratio. AT(N) 2 patients were positive for Aβ42 or the Aβ42/40 ratio and P-tau. AT(N) 3 patients had positive results for all three biomarkers. AT(N) 4 patients were positive for Aβ42 or the Aβ42/40 ratio and T-tau. Finally, AT(N) 5 patients were negative for Aβ42 or the Aβ42/40 ratio but positive for P-tau or T-tau or both biomarkers. For Lumipulse and Elecsys assays, classification was made based on the cut-offs provided by the manufacturers. The cut-offs for the Innotest assay were determined in an independent cohort of patients and controls in our lab. Our results indicated that although AT(N) classification based on the Aβ42/40 had the best discriminating power to correctly separate AD patients from non-AD patients with dementia (AUC 0.798; 95% CI 0.649–0.947), there were no significant differences between the four AT(N) classifications [i.e., Innotest, 2 lumipulse and Elecsys biomarkers based on the AT(N) classifications] with respect to diagnostic accuracy after comparing AUCs with the Hanley and McNeil method (|z| < 1.96). However, the sensitivity, specificity, and total percentage of predictive accuracy were different between methods, especially between Lumipulse and Innotest or Elecsys (Table 3). Among the three methods, Lumipulse AT(N)s had the best sensitivity (91.2%) and total predictive accuracy, while Elecsys AT(N) had the best specificity (77.8%) for discriminating AD from non-AD dementia patients.

**TABLE 3:** Diagnostic accuracy of the AT(N) classification for each method.

	**AT(N)**	**AUC (95% CI)**	**Sensitivity**	**Specificity**	Total% of predictive accuracy*	*z*-value**
Lumipulse	Aβ42/40, P-tau, (T-tau)	0.798 (0.649–0.947)	91.2%	71.4%	83.6%	*z* = 0.432 vs. Lumipulse Aβ42, P-tau, (T-tau); 0.288 vs. Innotest; *z* = 0.307 vs. Elecsys
	Aβ42, P-tau, (T-tau)	0.778 (0.617–0.939)	91.2%	71.4%	83.6%	*z* = −0.076 vs. Innotest; *z* = −0.034 vs. Elecsys
Innotest	Aβ42, P-tau, (T-tau)	0.783 (0.627–0.938)	79.4%	76.2%	78.2%	*z* = −0.052 vs. Elecsys
Elecsys	Aβ42, P-tau, (T-tau)	0.780 (0.624–0.937)	67.6%	77.8%	67.3%	

AUC, Area under the curve.

*The percentage of correct classification of AD + correct classification of non-AD/all cases.

**Values of | z| < 1.96 were taken as evidence that the true ROC areas were not different.

### CSF Biomarker Cut-Offs Based on Aβ42/40 Ratio Status

**FIGURE 4:**
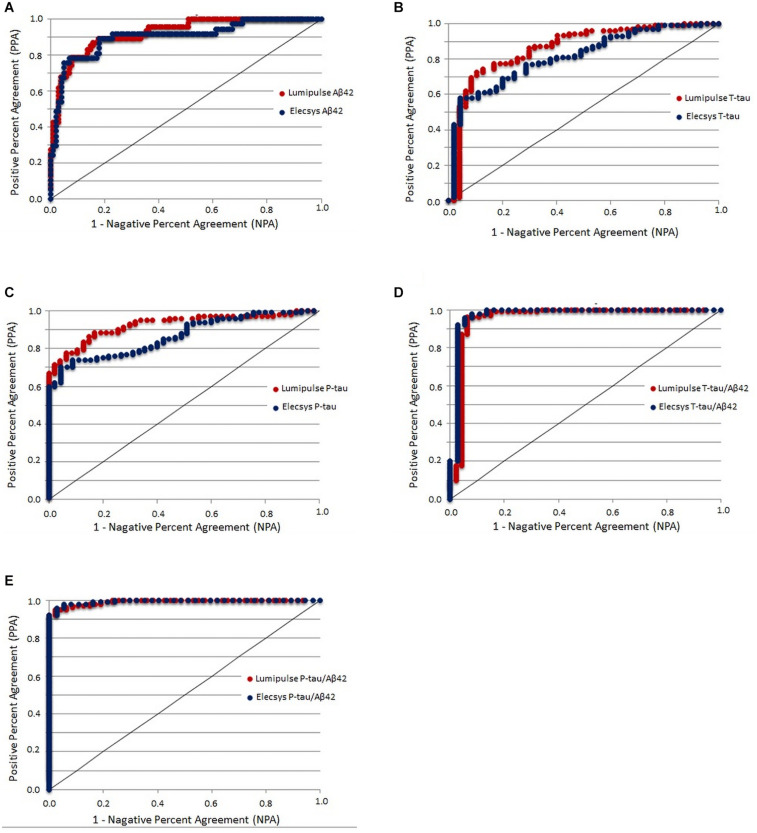
CSF biomarkers that yielded the maximum Youden index versus Aβ42/Aβ40 ratio status in the receiver operating characteristics analysis.

As the Aβ42/40 ratio and AT(N) had the best diagnostic accuracy, we selected these variables to serve as references for determining the cut-offs of biomarkers and ratios for Lumipulse and Elecsys. The cut-offs for each biomarker or ratio were established to be values that optimized the concordance with Aβ42/40 status as positive/negative. The determined cut-offs in this study and the established cut-offs by Fujirebio and Roche Diagnostics are presented in Table 4. As displayed in Table 4, the cut-offs determined according to concordance with the Aβ42/40 ratio were comparable with the manufacturer cut-offs. For Lumipulse and Elecsys biomarkers and ratios, the AUC for discriminating Aβ42/40 status was greater than 0.8. To discriminate between Aβ42/40 positivity/negativity status among Lumipulse assays, the Aβ42/40 AUC was 100, as it was used for calculating the cut-offs. However, the P-tau/Aβ42 and T-tau/Aβ42 ratios had a high discriminating accuracy (AUC 0.922, OPA 95.9% and AUC 0.956, OPA 95.3%, respectively) at the cut-off values of ≥0.082 and ≥0.517, respectively. Among Elecsys markers, the P-tau/Aβ42 and T-tau/Aβ42 ratios had superior discriminating power (AUC 0.994, OPA 96.4% and AUC 0.974, OPA 95.6%, respectively) at the cut-off values of ≥0.023 and ≥0.26, respectively. In fact, the Elecsys P-tau/Aβ42 was the best in discriminating patients based on Aβ42/40 status (Table 4 and Figure 4).

**TABLE 4:** Cut-offs of CSF biomarkers that yielded maximum Youden index versus Aβ42/Aβ40 ratio status in the receiver operating characteristics analysis.

		**AUC (95% CI)**	**PPA**	**NPA**	**Max Youden index**	**Cut-off**	**OPA**	**Manufacturer cutoffs**
Lumipulse	T-tau	0.860 (0.791–0.930)	72.5%	89.4%	61.9%	≥399	77.9%	>400
	P-tau	0.925 (0.884–0.967)	86.3%	85.1%	71.4%	≥51	85.9%	>56.5
	Aβ42	0.923 (0.878–0.967)	82.4%	89.4%	71.7%	≤563	84.6%	<600
	Aβ42/40	1.000 (1.000–1.000)	100%	100%	100%	≤0.070	100%	<0.069
	P-tau/Aβ42	0.992 (0.984–1.000)	95.1%	97.9%	93.0%	≥0.082	95.9%	–
	T-tau/Aβ42	0.956 (0.906–1.000)	96.1%	93.6%	89.7%	≥0.517	95.3%	–
Elecsys	T-tau	0.812 (0.739–0.885)	58.0%	95.6%	53.6%	≥268.15	69.7%	>300
	P-tau	0.867 (0.811–0.923)	70.0%	95.6%	65.6%	≥22.175	78.0%	>27
	Aβ42	0.904 (0.840–0.967)	93.0%	78.4%	71.4%	≤939.150	89.1%	≤1000
	P-tau/Aβ42	0.994 (0.986–1.000)	96.0%	97.3%	93.3%	≥0.023	96.4%	>0.024
	T-tau/Aβ42	0.974 (0.932–1.000)	96.0%	94.6%	90.6%	≥0.26	95.6%	>0.28

*AUC, Area under the curve; PPA, Positive percent agreement with Aβ42/40 status; NPA, Negative percent agreement with Aβ42/40 status; Max Youden index, (PPA + NPA – 1); OPA, Overall percent agreement.*

In addition, we determined the cut-offs for biomarkers and ratios of Lumipulse and Elecsys based on the Aβ42 status of Innotest (Supplementary Table 1). Among Lumipulse assays, Aβ42 had the highest AUC (0.955, 95% CI 0.924–0.986) followed by P-tau/Aβ42 (AUC 0.933, 95% CI 0.891–0.976) and Aβ42/40 (AUC 0.931, 95% CI 0.892–0.970). For the Elecsys assay, Aβ42 had the highest AUC (0.974, 95% CI 0.954–0.995), followed by P-tau/Aβ42 (AUC 0.936, 95% CI 0.894–0.978) and T-tau/Aβ42 (AUC 0.920, 95% CI 0.868–0.971).

Furthermore, all statistical analysis was performed for the study cohort separated by sex (Supplementary Figures 1–8 and Supplementary Tables 2–7). We found a high correlation between the three methods for both male and female subjects, although there were systematic differences between biomarker values measured by each method (Supplementary Figures 1–6). Interestingly, the cut-offs of CSF biomarkers for male subjects was lower than those of manufacturer, while in the case of female subjects these cut-offs were comparable with corresponding manufacturer’s cut-offs (Supplementary Tables 6, 7).

## Discussion

In this study, we evaluated the concordance between three different methods for measurement of AD CSF biomarkers—Innotest ELISA, Elecsys and Lumipulse platforms—in a cohort of patients with AD, MCI, and non-AD dementias. We also evaluated the diagnostic accuracy of biomarkers and their ratios measured by each method. Furthermore, we determined cut-offs for CSF biomarkers of AD (Aβ42, T-tau, and P-tau) and their ratios measured on the fully automated Lumipulse and Elecsys to optimize their concordance with Aβ42/40 status.

Although there was a high correlation between all three assays, our results showed that there was a lack of consistency between the three methods, except for Aβ42 and T-tau of Lumipulse and Innotest. Because the antibodies used for the Lumipulse assays were produced by the same manufacturer as Innotest ELISA (Fujirebio), the similar specificity of the antibodies between the two methods may partly explain the concordance we observed between Aβ42 and T-tau values between these two methods. Our results were consistent with previous studies that had found a systematic bias between the measurements of biomarkers by Lumipulse and Innotest (Bayart et al., 2019) and by Elecsys and Innotest (Willemse et al., 2018). The lack of concordance between Elecsys, Lumipulse, and Innotest assays may be attributed to the differences that exist between these methods. First, they have different recommended pre-analytical procedures that can affect the measured concentration of CSF biomarkers. Among these three biomarkers, Aβ42 is known to be more sensitive to pre-analytical conditions. Second, these methods use different measurement technologies (ECLIA, CLEIA, and ELISA, respectively), which may affect the detectable concentration. Third, the antibodies that were produced and applied in the AD CSF assays by Roche Diagnostics and Fujirebio Diagnostics may have different specificities. Finally, although both Elecsys and Lumipulse have been standardized for Aβ42, the material used for standardization differed between methods (Bittner et al., 2016; Kuhlmann et al., 2017).

We examined the ability of Aβ42, T-tau, P-tau and their ratios to discriminate AD patients from patients with non-AD dementias. Aβ42 and P-tau combined were both the best biomarkers for discriminating between the two diagnostic groups. Both biomarkers were specific to AD; therefore, it was not surprising that their combination had a high discriminating power for diagnosing AD patients. However, abnormal concentrations of T-tau in CSF, which underlies neurodegeneration, is not specific to AD and occurs in non-AD dementias or in non-AD elderly persons with comorbidities (Kovacs et al., 2013). Evaluation of differences in AUC revealed that there were no significant differences in the discriminating power of Aβ42 + P-tau measured by each method. However, the Innotest Aβ42 + P-tau had a better sensitivity (94%), specificity (76%), and predictive accuracy (87%). The Aβ42/40 ratio also had a high discriminating power for differentiating between patients with AD and non-AD dementias. Consistent with our results, Shoji et al. (1998) and Lewczuk et al. (2004) previously suggested that the Aβ42/40 ratio is superior to the concentration of Aβ42 alone for discriminating AD patients.

We also assessed the discriminating power of AT(N) groups that were generated by the results of CSF Aβ42 (Aβ42/40), P-tau and T-tau for each method. The AT(N) classification was proposed by the NIAA research framework (Jack et al., 2018) and gives a biological rather than a clinical definition of AD. We found that the use of Aβ42/40 instead of Aβ42 in AT(N) improved the classification accuracy (AUC 0.798, 95% CI 0.649–0.947 vs. AUC 0.778, 95% CI 0.617–0.939), However, the sensitivity, specificity and predictive accuracy was the same for both AT(N)s. Among all four AT(N) classifications, Elecsys AT(N) had the highest specificity. In fact, Elecsys AT(N) had better specificity than sensitivity in discriminating the two diagnostic groups. The preference for higher sensitivity or specificity depends on the purpose of different investigation scenarios. For example, for screening purposes, higher sensitivity is always preferable; however, high specificity might be preferable for the selection of patients for clinical trials. These results should be interpreted with caution because of the small population size of both of the diagnostic groups in our study.

Finally, we defined the CSF cut-offs for both Lumipulse and Elecsys assays based on the Lumipulse Aβ42/40 status because of its high diagnostic accuracy in our study, its high stability with respect to pre-analytical variations (Lewczuk et al., 2006; Willemse et al., 2018) and the fact that the ratio probably accounts for inter-individual variability in overall Aβ production and CSF turnover (Janelidze et al., 2017). Given that Innotest assays are among some of the most commonly used methods for the detection of AD CSF biomarkers, we also provided the cut-offs for both Lumipulse and Elecsys assays based on the Innotest Aβ42 status.

In previous studies, amyloid PET visual read (Schindler et al., 2018; Alcolea et al., 2019) or diagnostic accuracy (Bayart et al., 2019) have been used for the determination of AD CSF biomarkers cut-offs for fully automated methods and their ratios. Our results indicated that the cut-offs based on the Aβ42/40 ratio had a close similarity to the cut-offs established by each manufacturer; therefore, the Aβ42/40 ratio is a robust variable that can differentiate AD from non-AD individuals. Based on our results for both the Lumipulse and the Elecsys methods, P-tau/Aβ42 and T-tau/Aβ42 performed better together than each biomarker alone in discriminating Aβ42/Aβ40 ± status. This result is consistent with the results of previous studies where P-tau/Aβ42 (Alcolea et al., 2019) or T-tau/Aβ42 (Bayart et al., 2019) demonstrated superior performance in discriminating the diagnostic groups or amyloid PET status compared with individual biomarkers (Schindler et al., 2018).

Some limitations of this study require consideration. First, our study population lacked health control individuals. The majority of the population consisted of MCI subjects (*n* = 94) with a short follow-up time; for this reason, we decided to eliminate patients in some analyses and retain a small number of AD (*n* = 34) and non-AD demented patients (*n* = 21) when evaluating the diagnostic accuracy of biomarkers. Second, instead of using an independent method, we used the Aβ42/Aβ40 ratio status or Aβ42 status to determine the biomarker cut-offs, and this may have led to the overfitting of the results. Third, Aβ40 cannot be measured by Elecsys or ELISA, so, the comparison was incomplete. Other limitation is that 17 patients were excluded of the analyses because they had Aβ42 values above the upper limit of detection (1700 pg/ml) for Elecsys.

The main strength of our study is that we compared, for the first time, the clinical and analytical performance of fully automated Elecsys and Lumipulse platforms together in the same cohort of patients. In addition, our study population consisted of a real population of patients who attended a memory clinic and, therefore, provided a more realistic application of biomarkers in daily clinical practice.

## Conclusion

In conclusion, both Lumipulse and Elecsys methods had a high correlation with each other and with Innotest ELISA. The presence of systematic bias between biomarkers measured by each method was expected as there were various pre-analytical and analytical differences between methods. For both Lumipulse and Elecsys methods, ratios had a better analytical performance compared with individual biomarkers, and the Aβ42/Aβ40 ratio had a high concordance with the diagnostic accuracy of AD. Because the calibrators were adjusted with reference samples in both automated platforms, it was expected that these platforms would reduce intra- and inter-laboratory variations and enhance reproducibility.

## Author’s Note

Considering the importance of study of cerebrospinal fluid biomarkers in mild cognitive impairment and Alzheimer’s disease, we aimed to investigate the concordance between core AD biomarkers measured in CSF using Innotest, Lumipulse and Elecsys methods. We observed that both, Lumipulse and Elecsys methods had a high correlation with each other and with Innotest ELISA. The presence of systematic bias between biomarkers measured by each method was expected as there are various pre-analytical and analytical differences between methods. For both Lumipulse and Elecsys methods, ratios had a better analytical performance compared with individual biomarkers. The Lumipulse and Elecsys CSF AD assays showed high analytical and clinical performances so their use is recommended for the measurement of CSF AD biomarkers compared with unstandardized manual methods.

## Data Availability Statement

The raw data supporting the conclusions of this article will be made available by the authors, without undue reservation.

## Ethics Statement

The studies involving human participants were reviewed and approved by the Comite Etica Hospital Arnau Vilanova Lleida. The patients/participants provided their written informed consent to participate in this study.

## Author Contributions

FD, RL-O, and GP-R designed the study, analyzed the data, interpreted the data, and wrote the manuscript. IR-L, AA, RH, NT, and MR-J collected the data. All authors revised the manuscript and approved it for submission.

## Conflict of Interest

The authors declare that the research was conducted in the absence of any commercial or financial relationships that could be construed as a potential conflict of interest.
