# Supplementary material for: Assessment of the Concordance and Diagnostic Accuracy Between Elecsys and Lumipulse Fully Automated Platforms and Innotest
Source: Front Aging Neurosci. 2021 Mar 4;13:604119. doi: 10.3389/fnagi.2021.604119 (PMC7970049; doi:10.3389/fnagi.2021.604119)
Supplement: Supplementary file 1 [file Data_Sheet_1.docx]

Assessment of the concordance and diagnostic accuracy between Elecsys and Lumipulse fully automated platforms and Innotest

Automated AD CSF biomarkers analysis

Farida Dakterzada^1*^, Ricard López-Ortega^1*^, Alfonso Arias^1*^, Iolanda Riba-Llena^1*^, Maria Ruiz-Julián^1*^, Raquel Huerto^1*^, Nuria Tahan^1*^, Gerard Piñol-Ripoll^1**^

(1) Cognitive Disorders Unit, Clinical Neuroscience Research group, Santa Maria University Hospital, IRBLleida, Lleida, 25198, Spain.

* Co-first authors. FD and RLO contributed equally to this study.

****Corresponding author:**

Gerard Piñol Ripoll

Cognitive Disorders Unit

Hospital Universitari Santa Maria

Rovira Roure nº 44, 25198, Lleida, Spain

Telephone: 34-937-727222 Ext. 173. Fax: 34-976-727366

E-mail: [gerard_437302@hotmail.com](mailto:gerard_437302@hotmail.com)

Number of pages: 29

Number of words abstract (237), introduction (597), discussion (1498).

**Supplementary Table 1.** Cut-offs of CSF biomarkers that yielded the maximum Youden index versus Innotest Aβ42 status in the receiver operating characteristics analysis.

**Supplementary Figure 1.** The correlation and Bland-Altman plots for Aβ42 (n=65), T-tau (n=66) and P-tau (n=65) measurements obtained by Lumipulse and Innotest ELISA methods including only male subjects. Each point is defined as the measurements of Lumipulse and ELISA assays on the same biological sample. The correlation coefficient between the two methods was 0.88 for Aβ42 (P < 0.0001), 0.92 for T-tau (P < 0.0001) and 0.95 for P-tau (P < 0.0001). In the Bland-Altman plots, solid lines represent the slope observed. In this plot, the bias (mean of the differences) for Aβ42 was 17.30 units (pg/mL) for T-tau, 6.85 units and for P-tau, -11.07 units (continuous line) between two methods.


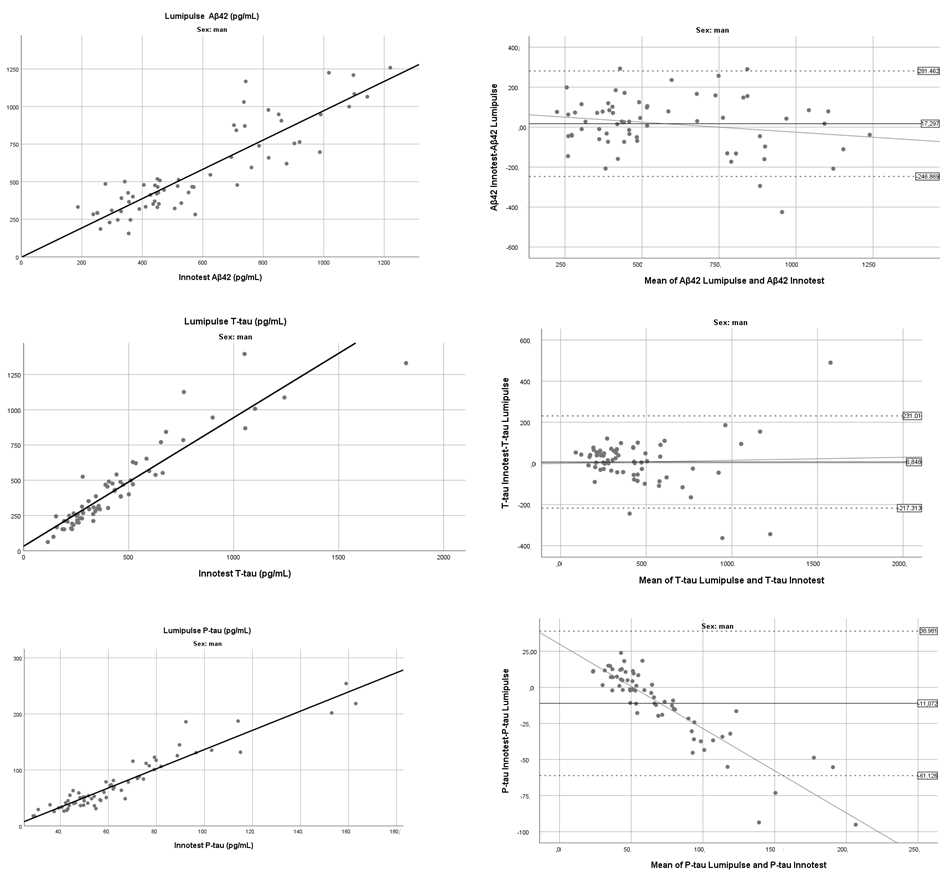


**Supplementary Figure 2.** The correlation and Bland-Altman plots for Aβ42 (n=82), T-tau (n=83) and P-tau (n=83) measurements obtained by Lumipulse and Innotest ELISA methods including only female subjects. Each point is defined as the measurements of Lumipulse and ELISA assays on the same biological sample. The correlation coefficient between the two methods was 0.85 for Aβ42 (P < 0.0001), 0.96 for T-tau (P < 0.0001) and 0.95 for P-tau (P < 0.0001). In the Bland-Altman plots, solid lines represent the slope observed. In this plot, the bias (mean of the differences) for Aβ42 was 6.58 units (pg/mL) for T-tau, -9.96 units and for P-tau, -16.87 units (continuous line) between two methods.


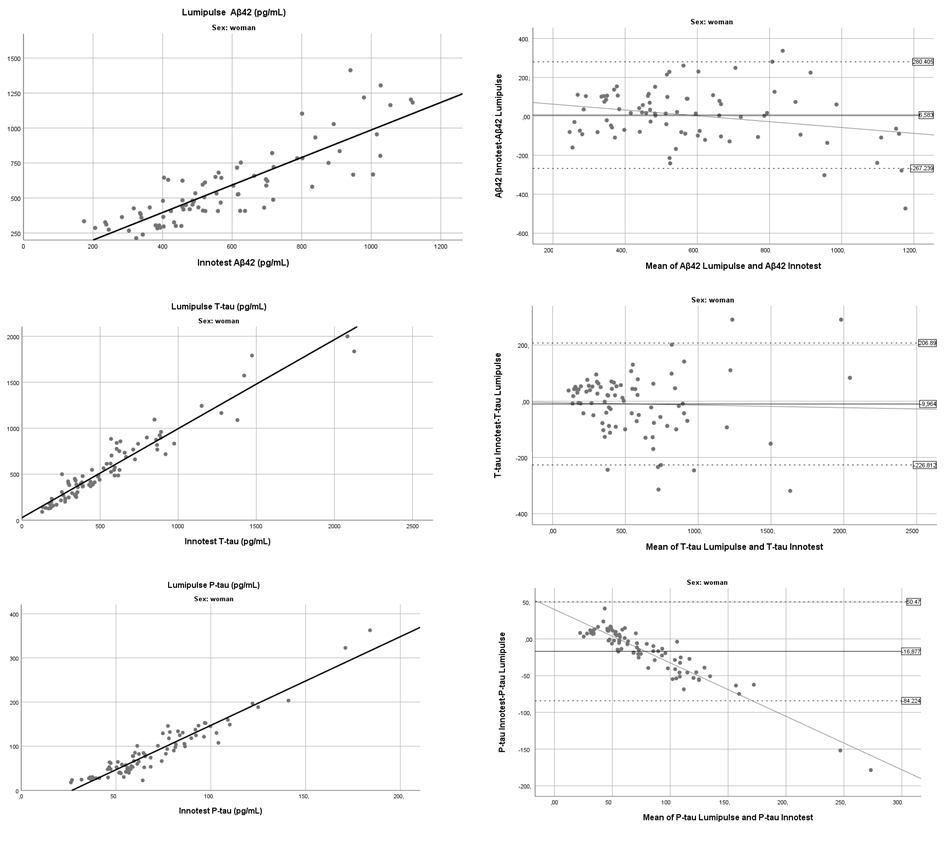


**Supplementary Figure 3.** The correlation and Bland-Altman plots for Aβ42 (n=59), T-tau (n=63) and P-tau (n=63) measurements obtained by Lumipulse and Elecsys methods including only male subjects. Each point is defined as the measurement of Lumipulse and Elecsys assays on the same biological sample. The correlation coefficient between the two methods was 0.94 for Aβ42 (P < 0.0001), 0.95 for T-tau (P < 0.0001) and 0.95 for P-tau (P < 0.0001). In the Bland-Altman plots, solid lines represent the slope observed. In this plot, the bias (mean of the differences) for Aβ42 was 240.85 units (pg/mL) for T-tau, -167.67 units and for P-tau, -48.94 units (continuous line) between two methods.


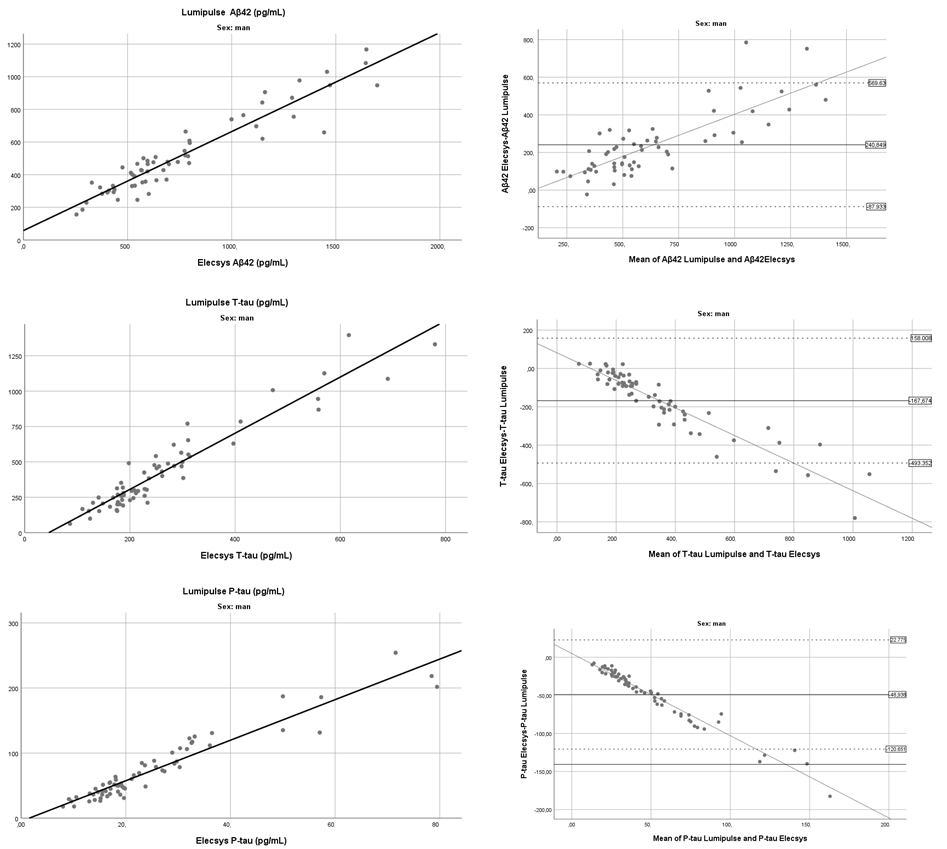


**Supplementary Figure 4.** The correlation and Bland-Altman plots for Aβ42 (n=78), T-tau (n=82) and P-tau (n=82) measurements obtained by Lumipulse and Elecsys methods including only female subjects. Each point is defined as the measurement of Lumipulse and Elecsys assays on the same biological sample. The correlation coefficient between the two methods was 0.93 for Aβ42 (P < 0.0001), 0.96 for T-tau (P < 0.0001) and 0.97 for P-tau (P < 0.0001). In the Bland-Altman plots, solid lines represent the slope observed. In this plot, the bias (mean of the differences) for Aβ42 was 245.12 units (pg/mL) for T-tau, -243.85 units and for P-tau, -58.11 units (continuous line) between two methods.


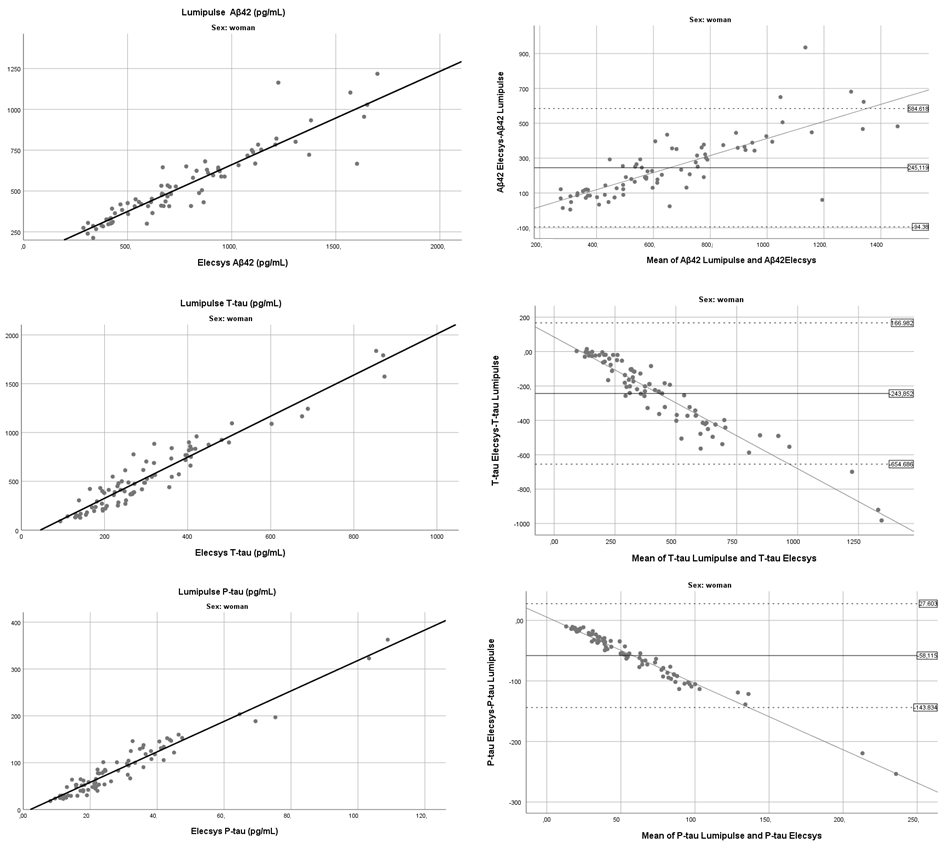


**Supplementary Figure 5.** The correlation and Bland-Altman plots for Aβ42 (n=59), T-tau (n=63) and P-tau (n=63) measurements obtained by Innotest ELISA and Elecsys methods including only male subjects. Each point is defined as the measurement of Innotest ELISA and Elecsys assays on the same biological sample. The correlation coefficient between the two methods was 0.87 for Aβ42 (P < 0.0001), 0.96 for T-tau (P < 0.0001) and 0.97 for P-tau (P < 0.0001). In the Bland-Altman plots, solid lines represent the slope observed. In this plot, the bias (mean of the differences) for Aβ42 was -217.28 units (pg/mL) for T-tau, 182.01 units and for P-tau, 38.89 units (continuous line) between two methods.


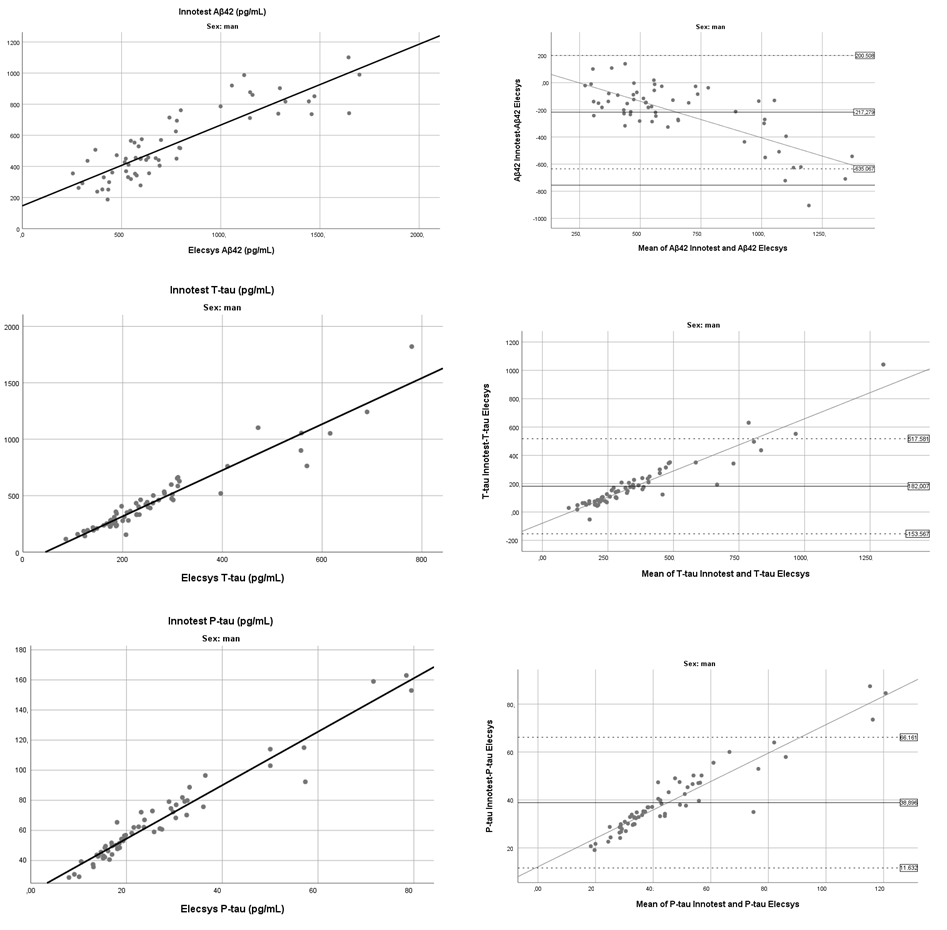


**Supplementary Figure 6.** The correlation and Bland-Altman plots for Aβ42 (n=78), T-tau (n=82) and P-tau (n=82) measurements obtained by Innotest ELISA and Elecsys methods including only female subjects. Each point is defined as the measurement of Innotest ELISA and Elecsys assays on the same biological sample. The correlation coefficient between the two methods was 0.88 for Aβ42 (P < 0.0001), 0.96 for T-tau (P < 0.0001) and 0.96 for P-tau (P < 0.0001). In the Bland-Altman plots, solid lines represent the slope observed. In this plot, the bias (mean of the differences) for Aβ42 was -225.78 units (pg/mL) for T-tau, 232.75 units and for P-tau, 41.12 units (continuous line) between two methods.


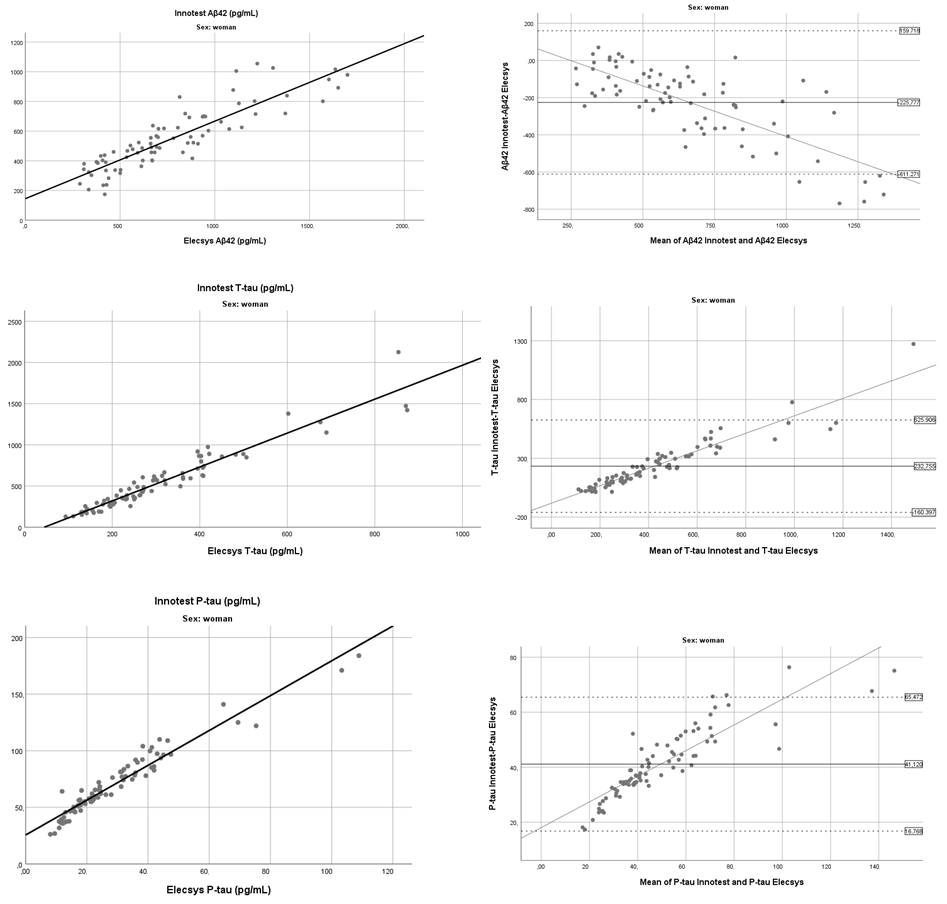


**Supplementary Figure 7.** CSF biomarkers that yielded the maximum Youden index versus Aβ42/Aβ40 ratio status in the receiver operating characteristics analysis (Male subjects).


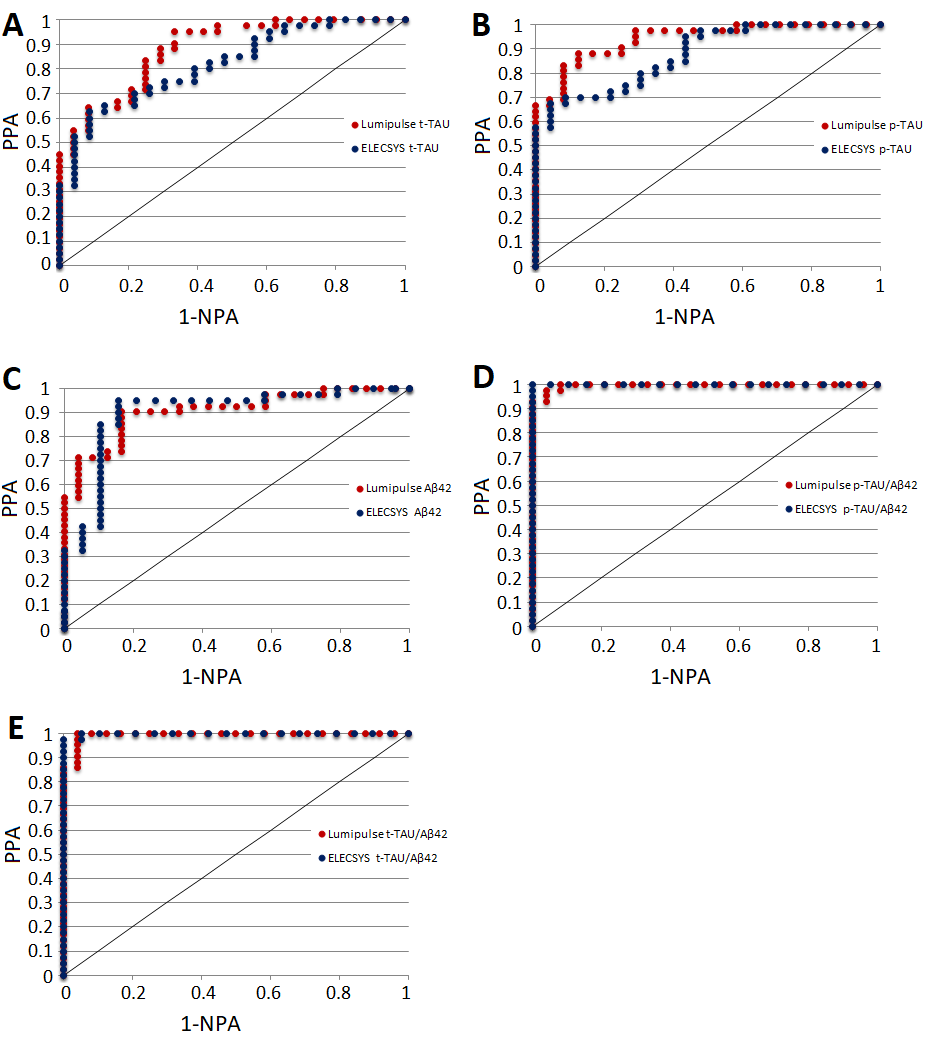


**Supplementary Figure 8.** CSF biomarkers that yielded the maximum Youden index versus Aβ42/Aβ40 ratio status in the receiver operating characteristics analysis (Female subjects).

**
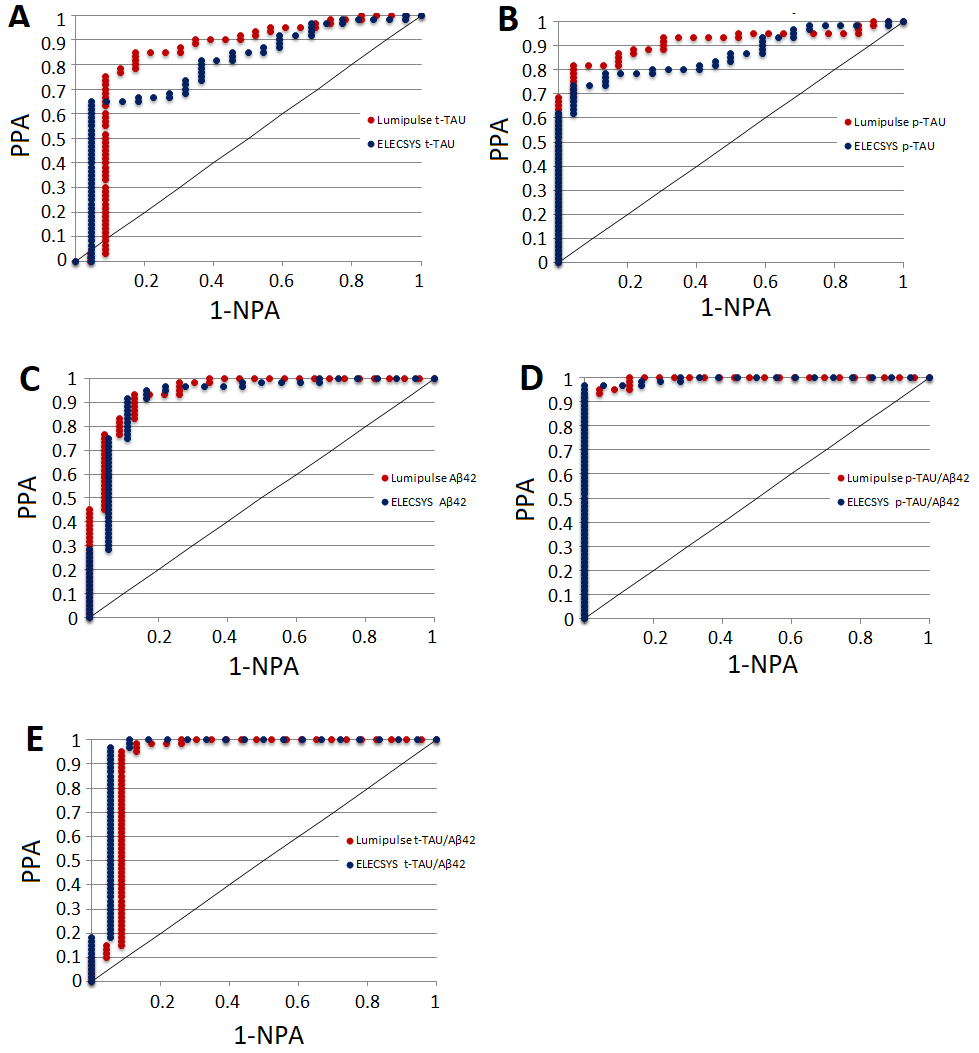
**

**Supplementary Table 2.** Biomarkers with the best discriminating power between AD and non-AD dementia patients (Male subjects).

**Supplementary Table 3.** Biomarkers with the best discriminating power between AD and non-AD dementia patients (Female subjects).

**Supplementary Table 4.** Diagnostic accuracy of the AT(N) classification for each method (Male subjects).

**Supplementary Table 5.** Diagnostic accuracy of the AT(N) classification for each method (Female subjects).

**Supplementary Table 6.** Cut-offs of CSF biomarkers that yielded the maximum Youden index versus Aβ42/Aβ40 ratio status in the receiver operating characteristics analysis (Male subjects).

**Supplementary Table 7.** Cut-offs of CSF biomarkers that yielded the maximum Youden index versus Aβ42/Aβ40 ratio status in the receiver operating characteristics analysis (Female subjects).
